# *Strongyloides* Pneumonia

**DOI:** 10.4269/ajtmh.2012.12-0108

**Published:** 2012-08-01

**Authors:** Juan Carlos Cataño, Miguel Alejandro Pinzón

**Affiliations:** Professors of Medicine, Infectious Diseases Section, Internal Medicine Department, University of Antioquia Medical School, Medellín, Colombia

**Figure 1.**
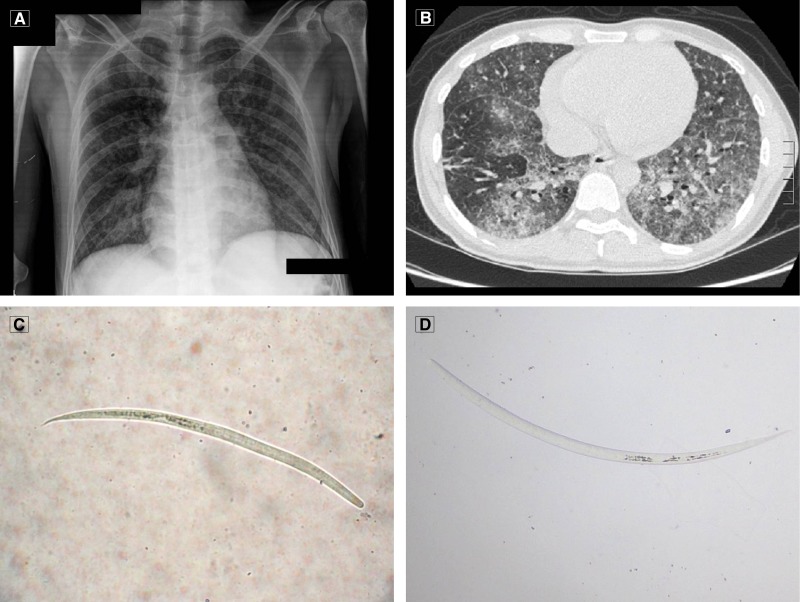
(**A**) Chest x-rays showing interstitial bilateral infiltrates. (**B**) High-resolution computed tomography scan showing bilateral ground-glass opacities. (**C**, **D**) Bronchoalveolar lavage with multiple *Strongyloides stercoralis* rhabditiform larvae.

A 32-year-old man presented to the emergency department with a 5-day history of dry cough, progressive dyspnea, nausea, and diarrhea. He gave no history of fever or other significant symptoms. The patient had human immunodeficiency virus (HIV) infection but was intermittently adherent to therapy. On examination, he appeared acutely ill, dehydrated, and cachectic. Vital signs showed a blood pressure of 100/60 mmHg, pulse 120/min, respiratory rate 49/min, and oxygen saturation 56% on room air, which improved to 92% with 50% oxygen by mask. Chest auscultation revealed occasional bilateral crackles. Liver was enlarged (span, 14 cm) but not tender. The remainder of the exam was normal. Laboratory data showed white blood cell 13.5 cells/mL (56% neutrophils, 5.7% eosinophils); hemoglobin 6.4 mg/dL, platelets 75,000/mL, creatinine 0.7 mg/dL, and CD4 count 17 cells/μL. Stool cultures for enteric pathogens were negative. Chest radiographs showed interstitial bilateral infiltrates (Figure 1A). A high-resolution computed tomography scan showed bilateral ground-glass opacities (Figure 1B). The patient was started empirically on intravenous trimethoprim-sulfamethoxazole and on hospital Day 2 a bronchoscopy was performed. The bronchoalveolar lavage revealed multiple *Strongyloides stercoralis* rhabditiform larvae (Figure 1C and 1D); all other tests, including stains for *Pneumocystis* and bacterial cultures were negative. On hospital Day 3 the patient developed respiratory failure, requiring transfer to the intensive care unit, mechanical ventilation, and one dose of ivermectin (200 mcg/kg PO); however, the patient developed disseminated intravascular coagulation with multisystem organ failure and expired on hospital Day 4.

